# Expression of intra- and extracellular granzymes in patients with typhoid fever

**DOI:** 10.1371/journal.pntd.0005823

**Published:** 2017-07-27

**Authors:** Hanna K. de Jong, Maria Isabel Garcia-Laorden, Arie J. Hoogendijk, Christopher M. Parry, Rapeephan R. Maude, Arjen M. Dondorp, Mohammed Abul Faiz, Tom van der Poll, Willem Joost Wiersinga

**Affiliations:** 1 Department of Internal Medicine, Division of Infectious Diseases and Center for Infection and Immunity Amsterdam (CINIMA), Academic Medical Center, Amsterdam, the Netherlands; 2 Center for Experimental and Molecular Medicine (CEMM), Academic Medical Center, University of Amsterdam, Amsterdam, the Netherlands; 3 Mahidol-Oxford Tropical Medicine Research Unit (MORU), Faculty of Tropical Medicine, Mahidol University, Bangkok, Thailand; 4 Center for Tropical Medicine, Nuffield Department of Clinical Medicine, Churchill Hospital, Oxford, United Kingdom; 5 Clinical Research Department, London School of Hygiene and Tropical Medicine, London, United Kingdom; 6 School of Tropical Medicine and Global Health, Nagasaki University, Nagasaki, Japan; 7 Chittagong Medical College Hospital, Chittagong, Bangladesh; 8 Dev Care Foundation, Dhaka, Bangladesh; International Vaccine Institute, REPUBLIC OF KOREA

## Abstract

**Background:**

Typhoid fever, caused by the intracellular pathogen *Salmonella* (*S*.) *enterica* serovar Typhi, remains a major cause of morbidity and mortality worldwide. Granzymes are serine proteases promoting cytotoxic lymphocytes mediated eradication of intracellular pathogens via the induction of cell death and which can also play a role in inflammation. We aimed to characterize the expression of extracellular and intracellular granzymes in patients with typhoid fever and whether the extracellular levels of granzyme correlated with IFN-γ release.

**Methods and principal findings:**

We analyzed soluble protein levels of extracellular granzyme A and B in healthy volunteers and patients with confirmed *S*. Typhi infection on admission and day of discharge, and investigated whether this correlated with interferon (IFN)-γ release, a cytokine significantly expressed in typhoid fever. The intracellular expression of granzyme A, B and K in subsets of lymphocytic cells was determined using flow cytometry. Patients demonstrated a marked increase of extracellular granzyme A and B in acute phase plasma and a correlation of both granzymes with IFN-γ release. In patients, lower plasma levels of granzyme B, but not granzyme A, were found at day of discharge compared to admission, indicating an association of granzyme B with stage of disease. Peripheral blood mononuclear cells of typhoid fever patients had a higher percentage of lymphocytic cells expressing intracellular granzyme A and granzyme B, but not granzyme K, compared to controls.

**Conclusion:**

The marked increase observed in extra- and intracellular levels of granzyme expression in patients with typhoid fever, and the correlation with stage of disease, suggests a role for granzymes in the host response to this disease.

## Author summary

Typhoid fever is an (sub)acute febrile illness that remains an important global burden with more than 27 million cases worldwide each year and an estimated 217,000 deaths. During infection by *Salmonella (S*.*)* Typhi, the etiologic agent for typhoid fever, a cascade of antimicrobial functions is triggered and causes release of signaling and cytotoxic proteins for the rapid control of the infection.

Granzymes are proteins promoting cytotoxic lymphocytes mediated eradication of intracellular pathogens via the induction of cell death and which can also play a role in inflammation. In the present study we analyzed extracellular levels of different granzymes in healthy volunteers and patients with confirmed *S*. Typhi infection at the time of admission and discharge, as well as their correlation with levels of interferon (IFN)-γ, a cytokine significantly expressed in typhoid fever. Patients demonstrated a marked increase of extracellular released granzyme A and B in acute phase plasma, which correlated with IFN-γ levels, while granzyme B levels were associated with disease stage. Intracellular expression of both granzymes was also increased in patients compared to controls. In conclusion, granzymes are markedly elevated in human typhoid and correlate with stage of disease, suggesting their involvement in the host response to the disease.

## Introduction

Typhoid fever, caused by the intracellular and human-specific Gram-negative bacterium *Salmonella (S*.*) enterica* serovar Typhi, remains an important cause of illness and death in many parts of the world. The global burden of this systemic infection is estimated to be around 26.9 million cases of per year resulting in over 200,000 deaths annually [1–3]. However, the remarkable mechanisms for cellular persistence of *S*. Typhi remain ill defined.

Cellular mediated immune responses against *S*. Typhi infection rely largely on two types of lymphocytic cells: CD4^+^ and CD8^+^ T cells [4]. The presence of both CD4^+^ helper T cells and *S*. Typhi-specific CD8^+^ T cells has been observed in humans challenged with oral *S*. Typhi or immunized with the attenuated oral Ty21a typhoid vaccine [4, 5]. Cytotoxic CD8^+^ T cells and natural killer (NK) cells are important effector cells of cell-mediated immunity and are involved in adaptive and innate immune responses. It is well established that T lymphocytes and NK cells are important sources of interferon (IFN)-γ, a critical cytokine for systemic control of *Salmonella* infection [6]. Interestingly, a human vaccine study showed that the killing of *S*. Typhi-infected cells by specific CD8^+^ T cells is executed through a Fas-independent, but granule-dependent mechanism, which suggests a role for granzymes in the containment of *S*. Typhi [7]. Granzymes are a family of serine proteases found in the cytoplasmic granules of cytotoxic lymphocytes. To date, five human granzymes (A, B, H, K and M) have been described of which granzyme A and B have been studied most extensively [8]. The classic role of granzymes is to promote cytotoxic lymphocytes-mediated eradication of infected, neoplastic, or foreign cells via the induction of cell death. However, it is now accepted that granzymes can be expressed in other cell types of immune and non-immune origin, and increasing evidence support that they can also play a role in inflammation [9]. Extracellular granzymes have been shown to exhibit different functions i.e. propagate inflammation and cytokine processing but not cell death [10]. Circulating granzymes can be measured in the plasma of patients and are considered to reflect the involvement of cytotoxic CD8^+^ T cells and NK cells in diverse disease states [11]. Elevated levels of granzyme B are a characteristic feature of various chronic inflammatory diseases and are thought to reflect a state of hyper-inflammation [10, 12]. Furthermore, it has been shown that granzymes are elevated in patients with malaria, endotoxemia, sepsis and tuberculosis [8, 12–14]. However, the expression of intra- and extracellular granzymes in patients with *S*. Typhi infection has, to the best of our knowledge, never been studied.

In the present study, we aimed to characterize the expression of extracellular and intracellular granzymes in patients with typhoid fever. For this, we analyzed the extracellular levels of granzyme A and B, as well as the intracellular expression of granzymes A, B and K in lymphocyte subsets, in patients with culture-proven typhoid fever compared to controls. We also investigated whether the extracellular levels of granzyme correlated with IFN-γ release.

## Materials and methods

### Patients and study design

A total of 143 eligible febrile adult patients were prospectively recruited over a 6-month period in 2012 after admission to Chittagong Medical College Hospital, a 1,000-bed government hospital located in Chittagong, eastern Bangladesh. From all these febrile patients, blood samples (24 mL) were obtained within 48 hours after admission to the hospital and at discharge, and were collected into EDTA or heparin tubes (BD vacutainer), or BactAlert blood culture bottles (bioMérieux). A total of 28 of the 143 febrile patients tested positive for *S*. Typhi either with blood-culture and/or *S*. Typhi PCR in blood, urine or feces as described [15].

From a subset of 8 patients with only blood-culture confirmed typhoid fever additional consent was required and blood was drawn (30 mL) within 72 hours and collected in CPT Cell Preparation Tubes with sodium heparin for cell separation (BD Vacutainer).

Thirty-eight healthy Bangladeshi volunteers from among the hospital staff who were known to have no illness and were not currently receiving any medication were recruited and served as control population.

### Ethics statement

The study protocol was approved by the National Research Ethics Committee (NREC) of Bangladesh (BMRC/NREC/2010-2013/1543) and the Oxford Tropical Research Ethics committee (OXTREC reference 25–11). Informed written or thumbprint consent was taken from the subject or caretaker for all cases and controls.

### Assays

Plasma samples were stored immediately at −20°C after obtention. Soluble granzyme A and B were measured by sandwich ELISA (eBioscience; LD 2 pg/ml) in plasma, in accordance with the manufacturer’s recommendations. Human tumour necrosis factor (TNF)-α, interleukin (IL)-1β, IL-6, IL-8, IL-10, IL-12p70, and IFN-γ were measured by cytometric-bead-array multiplex assay (BD Biosciences; LD 0.5 pg/mL). Aspartate transaminase (AST), alanine transaminase (ALT) and renal function were measured in plasma with spectrophotometry (Roche Diagnostics) as for previous study [16].

### Flow cytometry

Peripheral blood mononuclear cells (PBMCs) were isolated from blood collected in CPT Cell Preparation Tubes and handled according to manufacturer's instructions (BD Vacutainer). After isolation, PBMCs were counted using count chamber (Loptik labor) and cells were suspended in RPMI 1640 (Gibco) with 20% Fetal Bovine Serum (FBS; Lonza). Another equal volume of RPMI with 20% FBS and dimethyl sulfoxide (Gibco) was gently added to the cryovial drop by drop. To ensure stepwise temperature decrease, cryovials containing PBMCs were put in an alcohol-free container using a temperature exchange system (CoolCell, Biocision) to achieve temperature lowering at ~1°C/min rate and placed in a −80°C refrigerator before shipment. Prior to analysis stored cells were carefully thawed, washed and stained with monoclonal antibodies against CD3 (AF700), CD4 (PerCP-Cy5.5), CD56 (APC) (all from BD Pharmingen) and CD8 (PE-Cy7; Biolegend), at 4°C for 25 min in the dark. For the intracellular staining, cells were fixed for 20 min in Cytofix/Cytoperm (BD Bioscience) at 4°C in the dark before washing. Subsequently, the cells were suspended in a buffer containing the antibodies against granzyme A (PE; BD Pharmingen), granzyme B (PE-CF594; BD Horizon) and granzyme K (FITC; Santa Cruz Biotechnology) before analyzing with a FACSCanto (BD Bioscience). FlowJo software (Tree Star Inc.) was used for analysis. Lymphocytes were gated in the forward scatter versus side scatter dot plot. Cells were selected as CD3^+^ or CD56^+^, or as CD3^+^CD4^+^ (CD4^+^ T cells), CD3^+^CD8^+^ (CD8^+^ T cells), CD3^+^CD56^+^ (CD56^+^ T cells) and CD3^−^CD56^+^ (NK cells), and expression of granzymes was analyzed in these populations as described previously [14]. The results are expressed as percentage of cells of the specific lymphocyte population expressing the corresponding granzyme and as the median fluorescence intensity (MFI). Alternatively, to analyze the lymphocyte source of each granzyme, cells were selected as positive for each granzyme and the percentage of the above-mentioned lymphocyte subpopulations were analyzed within the granzyme-positive lymphocytes.

### Statistical analysis

Values are expressed as median and interquartile ranges (IQR) unless indicated otherwise. Differences between groups were analyzed by Mann-Whitney *U* test. For correlations Spearman Rho is reported. These analyses were performed using GraphPad Prism version 6.0 for Mac (GraphPad Software) and SPSS version 15.0 (Chicago, Ill, USA). A P<0.05 was considered to represent a statistically significant difference.

## Results

### Pro-inflammatory cytokine profile in patients with PCR or culture-proven typhoid fever

We included 28 patients with confirmed typhoid fever: 11 (39%) of these confirmed cases were diagnosed by isolation of *S*. Typhi from blood, and 17 (61%) by positive *S*. Typhi PCR in blood (15), urine (2) and/or feces (1) [15]. A summary of baseline clinical features and laboratory results of patients and controls are shown in Table 1. While the total white blood cell count (WBC) did not differ between patients and controls, there was a significant shift towards a higher neutrophil and lower lymphocyte count in typhoid fever patients, which is a characteristic clinical feature for this disease [17]. Consistent with the literature on typhoid fever [2], levels of IL-6, IL-8, IL12p70 and IFN-γ were significantly increased in patients compared to controls. However, IFN-γ was the only marker that was significantly increased when comparing blood-culture negative/PCR positive patients with blood-culture positive patients (median 2.0 IQR [0.5–156] versus 598 [292–773], P<0.001), suggesting a role for IFN-γ during active bacterial replication of *S*. Typhi in the blood stream. IL-10 tended to be elevated in patients but the difference with controls did not reach statistical significance, partly due to a large inter-individual variation. IL-1β was undetectable and TNF-α was low in both patients and controls.

**Table 1:** Demographic, clinical and laboratory features of typhoid fever patients compared to controls.

	Healthy controls (n = 38)	Patients (n = 28)
**Characteristic**		
Age (years)	34 (19–55)	28 (20–45)
Sex (male)	19 (50)	16 (57)
Fever duration before admission (days)	0 (0–1)	7 (1–15)***
Duration of admission (days)	0 (0–0)	5 (0–12)***
Death in hospital	0 (0–0)	1 (3.7)
Temperature (Celsius)	36.1 (33.8–37.4)	38.8 (38–40.1)***
Systolic BP (mmHg)	120 (106–169)	116 (85–159)*
Pulse (beats/min)	73 (40–120)	104 (70–150)***
BUN (mg/dL)	6–20^a^	12.5 (9.3–76)
Serum creatinine (mg/dL)	0.1–0.5^a^	0.9 (0.6–2.2)
AST (U/L)	<40^a^	40 (13–1430)
ALT (U/L)	<45^a^	49 (15–1199)
Hb (mmol/L)	13.3 (8.4–15.5)	12.1 (3.5–14.5)**
WBC count1 (x10^9^ cells/L)	7.5 (4.5–10)	9 (1.5–29)
Neutrophil (%)	57 (43–76)	77 (3–92)***
Monocyte (%)	3 (2–9.3)	2 (0–7)
Lymphocyte (%)	36 (20–50)	19 (1–36)***
**Cytokines (pg/ml)**		
TNF-α	3.8 (2.4–6.4)	3.8 (3.2–9.4)
IL-8	5.5 (3.3–10)	18.2 (5.1–96)***
IL-6	1.1 (0.5–3.4)	14.5 (2.0–255)***
IL-1β	ND	ND
IL-10	ND	0.5 (0.5–12.2)
IL-12	0.5 (0.5–2.0)	1.2 (0.5–2.5)*
IFN-γ	0.5 (0.5–4.5)	122.4 (0.5–1619)***

Proportions are expressed as number (%) and continuous variables as median (range). P values are determined via Mann-Whitney *U* tests comparing typhoid fever patients to healthy controls.

* P<0.05,

** P<0.01,

*** P<0.001,

^a^ Normal range, not measured in the control group. WBC: white blood cell count. ND: non detectable.

### Extracellular granzyme A and B levels are highly elevated in patients with typhoid fever

**Fig 1:**
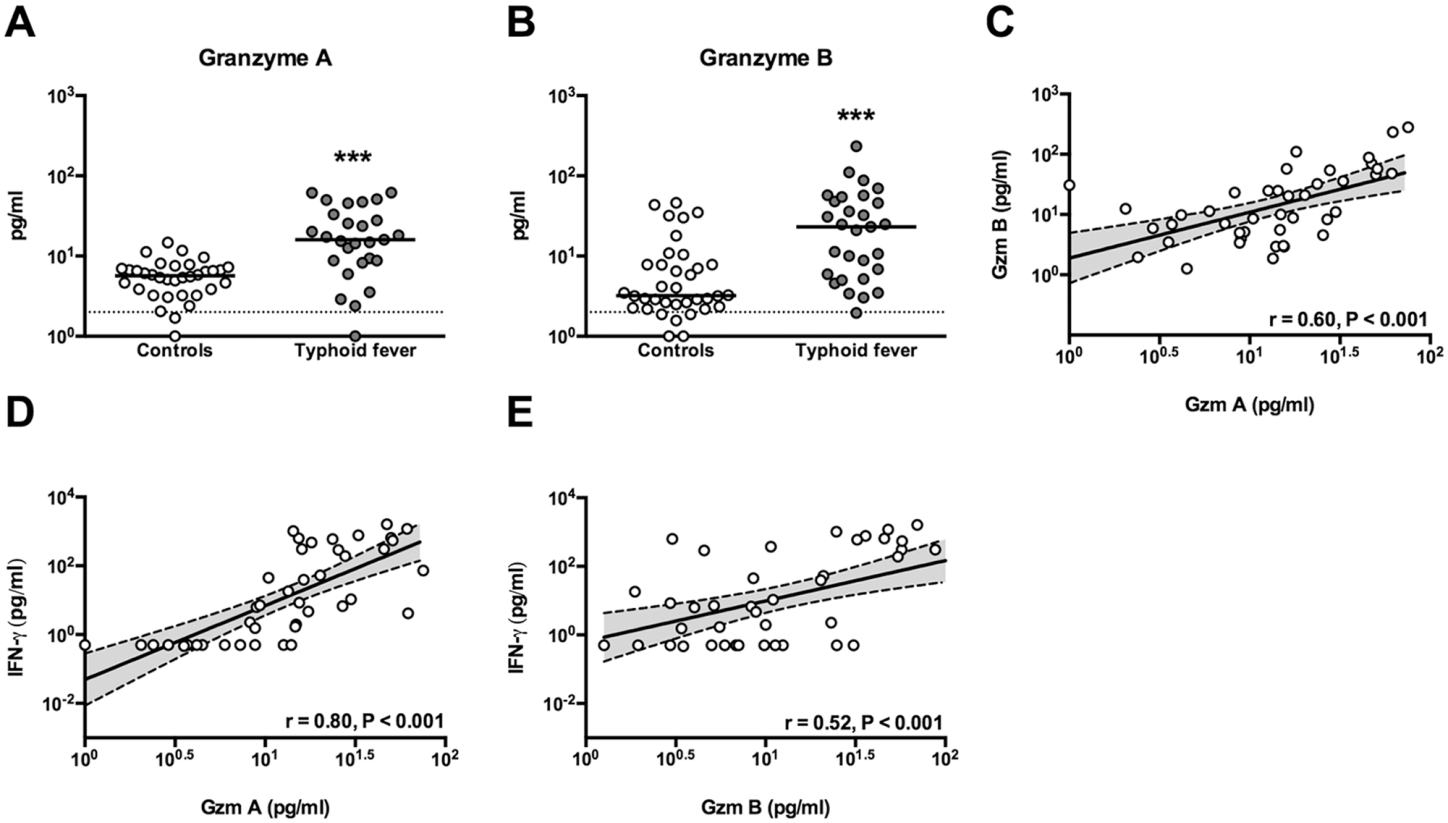
Extracellular levels of granzyme A and B in patients with typhoid fever and healthy controls, and correlation with IFN- γ levels. Plasma levels of granzyme A (A) and B (B) measured in healthy controls (n = 38) compared to admission samples of hospitalized typhoid fever patients (n = 28). Medians are shown. Significance determined via Mann-Whitney *U* tests. ***P<0.001. Granzyme A is correlated to granzyme B (C). Levels of granzyme A (D) and granzyme B (E) are correlated to interferon (IFN)-γ in patients. Correlation coefficient reported is for Spearman's Rho. Gzm: granzyme.

To first establish the presence of circulating granzymes during clinical typhoid fever we measured granzyme A and B in the plasma of 28 patients with *S*. Typhi infection and 38 healthy controls. Both granzyme A and B were elevated in the plasma of patients with typhoid fever compared to healthy controls (median 16 IQR [8.8–33] versus 5.7 [3.7–7.1] pg/ml, *P*<0.001, and median 23.3 IQR [5.5–51.3] versus 3.2, [2.4–7.8] pg/ml, *P*<0.001 respectively; Fig 1A–1B). In patients, a moderate correlation was seen between granzyme A and B levels (Spearman Rho r = 0.60; *P*<0.001, Fig 1C). Granzyme A levels (P<0.01), but not granzyme B levels (P = 0.16) were significantly elevated in culture-positive patients compared to culture negative/PCR-positive patients, which suggests that bacteria circulating in the bloodstream are inducers of extracellular granzyme A release. We next determined the correlation between extracellular granzymes and IFN-γ levels, since IFN-γ showed to be an important acute phase cytokine in this cohort. Positive correlations were observed between IFN-γ and granzyme A (Spearman´s Rho r = 0.80, P<0.001; Fig 1D), and granzyme B (Spearman Rho r = 0.52, P<0.001; Fig 1E).

### Plasma levels of granzyme B, but not granzyme A levels return to normal on day of discharge

**Fig 2:**
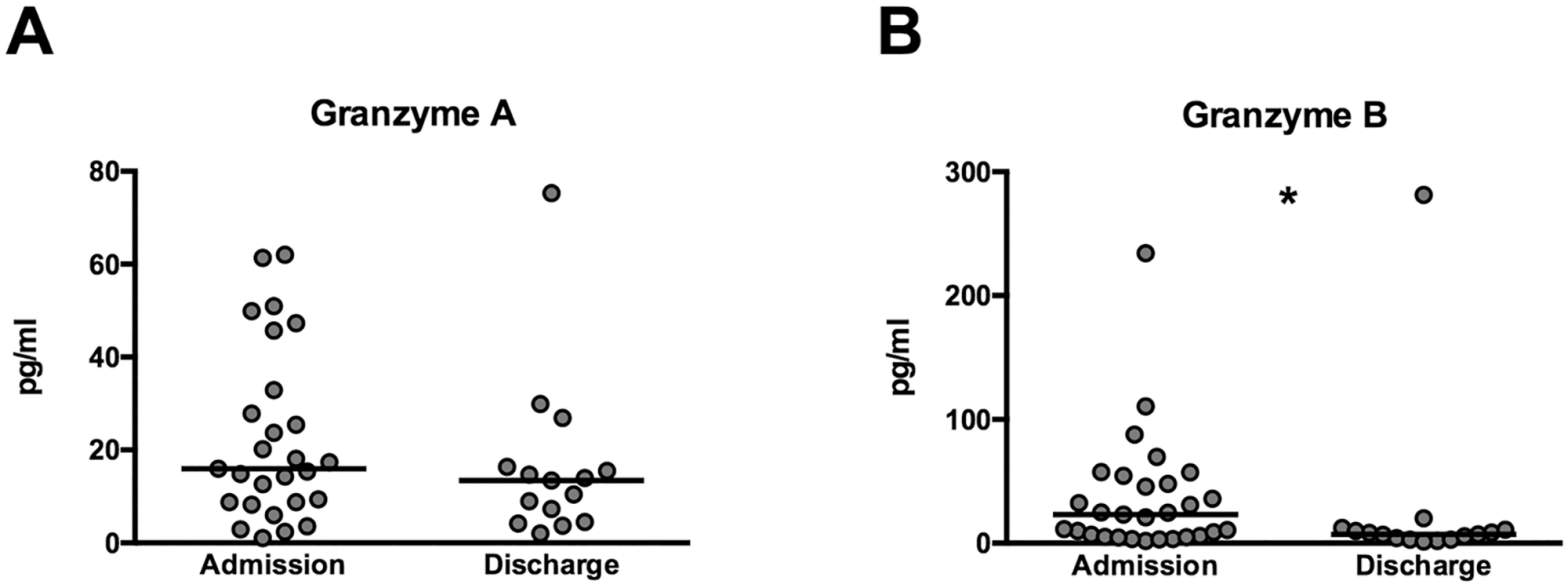
Extracellular levels of granzyme A and B on admission and during discharge in patients. Typhoid fever patients (n = 15) who were discharged from hospital had lower levels of granzymes at follow-up when patients were clinically improved (granzyme A; A), although this did only reach statistical significance for granzyme B (B). Medians are shown. Significance determined via Mann-Whitney *U* tests. *P<0.05. Gzm: granzyme.

In order to determine if granzyme levels correlated with stage of disease we obtained plasma samples of patients at discharge and compared them to admission samples. Plasma granzyme B (median 7.1, IQR [3–11] pg/ml, P<0.05), but not granzyme A levels (median 13.4, IQR [4.5–16.4] pg/ml, P = 0.26) were decreased at follow-up when patients were clinically improved (Fig 2A–2B). Plasma levels of granzyme B (P = 0.18), but not granzyme A (P<0.01) levels returned to normal during convalescence comparing samples of healthy controls to patient discharge samples.

### Lymphocytes of typhoid fever patients have a higher expression of intracellular granzymes than controls

To identify the lymphocytes subsets and the cells expressing intracellular granzymes A, B and K, flow cytometry was performed in cells from 36 control individuals and from 8 culture-positive typhoid fever patients. As in the total groups of controls and patients, in these subgroups both percentage of cells (median 34.5 IQR [31.5–39.3] and 17.5 [15.0–21.0] respectively for controls and patients; P<0.0001) and cell numbers (median 28.3 IQR [23.4–30.4] and 11.6 [8.1–15.5] cells x 10^8^/L respectively; P<0.0001) were lower in typhoid fever patients. A significant decrease in cell numbers of CD8^+^T, CD4^+^T, CD56^+^T and NK cells was found in patients compared to controls (Table 2). However, only CD4^+^ T cells presented a lower percentage in patients. Interestingly, the percentage of NK cells remained unchanged, and CD8^+^T as well as CD56^+^T cells, a subset of innate lymphocytes that possess the characteristics of both NK and T cells [18], showed higher percentages in the typhoid fever group (Table 2). In line with the measured extracellular granzyme levels, lymphocytes of typhoid fever patients had a higher percentage of cells expressing intracellular granzyme A and granzyme B than controls, although the increase in cell numbers was not significant (Table 2). We also measured the cells expressing granzyme K, which is thought to stimulate monocytic cells to secrete pro-inflammatory mediators like granzyme A [11], and this was increased in typhoid fever patients albeit not statistically significantly (Table 2). On day of discharge, percentage of total lymphocytes expressing granzymes remained comparable to day of admission and significantly different from controls (Table 2). In typhoid fever patients, the number of lymphocytes producing both granzyme A and B simultaneously was almost doubled compared to controls (25% vs 49%, P<0.001).

**Table 2:** Proportion and cell counts of lymphocyte populations and granzymes in patients with typhoid fever and controls.

	Controls	Typhoid fever	Discharge
Lymphocyte subsets			
CD8^+^ T cells			
Cells x 10^8^/L	5.8 [4.5–7.6]	2.9 [2.0–4.2]	N/A
%	20.7 [19.3–26.4]	26.8 [22.2–31.6]**	22.9 [20.2–24.8]
CD4^+^ T cells			
Cells x 10^8^/L	8.5 [6.8–11.5]	2.7 [1.2–3.2]***	N/A
%	33.5 [25.8–38.0]	18.0 [14.4–24.4]**	18.4 [13.0–24.4]
CD56^+^ T cells			
Cells x 10^8^/L	0.9 [0.6–1.3]	0.4 [0.1–0.9]	N/A
%	3.3 [2.6–4.8]	8.5 [6.3–10.9]*	6.1 [5.4–6.9]
NK cells			
Cells x 10^8^/L	3.1 [2.7–3.8]	0.7 [0.4–1.0]**	N/A
%	12.6 [8.8–16.4]	12.7 [8.6–17.2]	6.7 [6.3–7.1]
Granzyme A^+^ cells			
Cells x 10^8^/L	26.1 [22.3–33.7]	36.9 [26.8–43.9]	N/A
%	35.1 [29.6–41.4]	59.8 [53.6–62.2]***	60.6 [48.4–69.8]*
Granzyme B^+^ cells			
Cells x 10^8^/L	25.5 [22.6–37.0]	36.6 [29.3–54.5]	N/A
%	35.5 [29.3–41.4]	63 [57.2–71.6]***	70.8 [62.0–77.1]**
Granzyme K^+^ cells			
Cells x 10^8^/L	13.6 [10.9–15.9]	17.7 [10.9–19.8]	N/A
%	16.7 [14.6–21.9]	27.8 [18.2–31.8]	37.5 [31.4–43.7]**

Percentage values are percentages of total lymphocytes. Both cells counts and percentages are presented as median [IQR]. Analysis show control versus typhoid fever patients comparison by non-parametric test for two independent variables, Mann-Whitney *U* test.

*P<0.05,

**P<0.01,

***P<0.001 vs controls.

For all cells except CD56+: n = 36 for controls, 8 for typhoid admission, 4 for typhoid discharge.

For CD56+ cells: n = 25 for controls, 4 for typhoid admission, 2 for typhoid discharge.

N/A: WBC was not available on day of discharge, therefore total number of cells could not be measured.

We then analyzed which lymphocyte subsets were expressing each granzyme (that is, the cellular source). For this, we identified by flow cytometry the cells positive for each granzyme within the lymphocytes gate, and determined the percentage of cells belonging to each lymphocyte subset in each group of granzyme^+^ cells. As expected [14], we found that CD3^+^ cells were the main producers of the three granzymes, with a significant increase in the percentage of granzyme A and B expressed by CD3^+^ cells in patients compared to controls (median values of total cells positive for each granzyme that were CD3^+^, controls vs patients: 66% vs 87% for granzyme A, 53% vs 78% for granzyme B, and 91% vs 80% for granzyme K).

### Typhoid fever patients have higher percentages of all subsets of cells expressing granzyme A compared to controls

**Fig 3:**
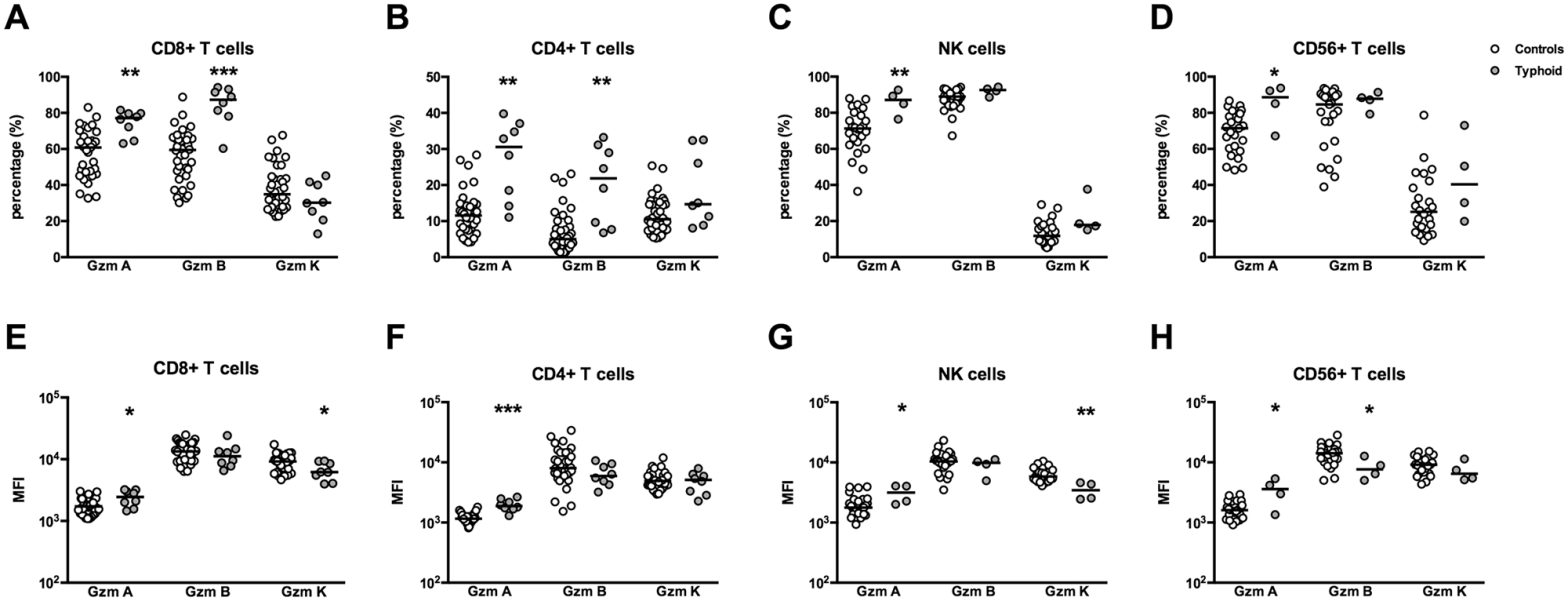
Expression of granzymes A, B and K by each lymphocyte subtype in patients with typhoid fever and controls. Percentage of lymphocyte population expressing each granzyme (A–D) and MFI of the intracellular expression of each granzyme in the lymphocyte populations (E–H). n = 36 controls and 8 patients. MFI values are expressed with Log10 scale. Significance determined via Mann-Whitney *U* tests. Medians are shown. Analysis was done by flow cytometry. MFI: median fluorescence intensity; NK cells: CD3^−^CD56^+^. *P<0.05, **P<0.01, ***P<0.001.

Next, we determined the percentage of cells that expressed granzymes per lymphocyte subset (Fig 3A–3D). For this, we identified by flow cytometry each lymphocyte subset within the lymphocytes gate, and determined the percentage of granzyme^+^ cells in each subset. We found that the percentage of CD8^+^ T cells, CD4^+^ T cells, CD56^+^ T cells, and NK cells positive for granzyme A was significantly higher in patients compared to controls. The percentage of CD8^+^ T cells and CD4^+^ T cells positive for granzyme B was also significantly higher in patients than controls. This was not the case for CD56^+^ T cells or NK cells. Percentages of subset of lymphocytes expressing granzyme K did not differ between both groups of individuals.

The median fluorescence intensity (MFI) of the three different granzymes (i.e., the amount of each granzyme expressed by granzyme^+^ cells) was also determined in each subset of lymphocytes (Fig 3E–3H). The MFI of cells expressing granzyme A was significantly increased in CD8^+^ T, CD4^+^ T, CD56^+^ T and NK cells, whilst the MFI of the CD56^+^ T cells expressing granzyme B was lower in patients compared to controls. The MFI of cells expressing granzyme K was also significantly lower in CD8^+^ T cells and NK cells in patients compared to controls.

## Discussion

Our study aimed to investigate the extracellular levels of granzymes A and B, as well as the intracellular expression of granzymes A, B and K in different lymphocyte subsets in patients with confirmed *S*. Typhi infection at the time of admission and discharge, and compare them to healthy controls. To the best of our knowledge, this is the first study to describe the expression of granzymes in patients with typhoid fever. Additionally, we intended to examine the correlation between granzymes expression and IFN-γ levels. The present study showed an increase in extracellular levels of granzyme A and B in patients with typhoid fever compared to controls, and lower levels of granzyme B in patients at day of discharge compared to admission. The extracellular expression of both granzymes correlated in patients with the concentration of IFN-γ, a critical cytokine for systemic control of *Salmonella* infection. On the other hand, the proportion of different subsets of lymphocytes expressing intracellularly granzyme A and granzyme B were increased in patients at acute stage.

Typhoid fever is a systemic infection in which lymphocytes play an essential role in the protective immune response [4, 19]. In contrast to other Gram-negative bacterial infections, typhoid fever patients usually have normal to low leucocyte numbers, and low lymphocyte counts [17]. In corroboration, patients reported in this study had a normal leucocyte count with markedly lower lymphocyte numbers, accompanied with a decrease in the percentage of CD4^+^ T cells but an increase of CD8^+^ T and CD56^+^T cells. IFN-γ is critical for the systemic control of *S*. *enterica* infections. Although CD4^+^ T cells were thought to be the key producers of IFN-γ during *Salmonella* infections [19,20], it is well established that T cells and NK cells are important sources of this cytokine [4]. Human challenge studies using attenuated *S*. Typhi live oral vaccines have shown that sensitized lymphocytes proliferate and produce IFN-γ in response to a number of *S*. Typhi-antigens [20]. CD4^+^ T cells are apparently the predominant IFN-γ-secreting cells associated with this response, although CD8^+^ T cells are also present and secrete IFN-γ [19, 21]. Unsurprisingly, we found elevated levels of IFN- γ in patients compared to controls.

Granzymes are serine proteases promoting cytotoxic lymphocytes-mediated eradication of intracellular pathogens via the induction of cell death [10]. Granzymes are delivered intracellularly into target cells where they can activate pathways of apoptosis. Furthermore, granzymes can be released into the extracellular environment by both immune and non-immune cells, and may propagate cytokine processing and inflammation, so having other functions than intracellular cytotoxicity [10]. Elevated levels of soluble granzymes have been reported in many different infectious diseases, reaching peak levels in patients with severe sepsis [11, 13]. In addition, we have demonstrated earlier that bacterial stimuli can induce the extracellular release of granzymes [11]. A study using PBMCs from individuals immunized with the Ty21a typhoid vaccine, has shown that CD8^+^ T cell-induced cytotoxicity was mediated by the granule exocytosis pathway [7], involving the release of perforin and granzymes into the intercellular space, thereby mediating cell death [8, 10]. Another report, however, provided results demonstrating that IFN-γ–independent cytotoxic mechanisms, mediated by granzymes or perforin [6], were not sufficient for NK cell—mediated limitation of the bacterial replication of *S*. Typhimurium. In the present study, we demonstrate that plasma concentrations of extracellular granzyme A and granzyme B were both elevated in typhoid fever at day of admission. Disease severity correlated with extracellular granzymes, as plasma granzyme correlated with IFN-γ levels and lower levels were seen on day of discharge compared to admission, which is in line with an earlier study using the typhoid vaccine [19]. This may suggest a role for granzymes in the stimulation of IFN-γ release, or that the inflammatory process simultaneously activates the release of both proteins. Of note, extracellular granzyme A, but not B, was significantly elevated in culture-positive patients suggesting that bacteria circulating in the bloodstream are potent inducers of extracellular granzyme A release.

When we explored the intracellular expression of granzyme A, B and K in lymphocytes by flow cytometry, we found that, in spite of the decreased numbers of lymphocytes, both the numbers (although non-significant) and the percentages of granzyme A and B, were increased in culture-proven typhoid fever patients compared to controls. As expected [14], we observed that the CD3^+^ lymphocytes were the main producers of the intracellular granzymes, both in controls and patients. In this study, we also found that all subsets of lymphocytes were expressing granzyme A in significantly higher percentages and with greater MFI (that is, the granzyme^+^ cells were expressing increased amounts of granzyme) in patients than in controls, suggesting a role for granzyme A during typhoid fever. For granzyme B, the percentage of CD8^+^T and CD4^+^T cells, but not CD56+T and NK cells, expressing the granzyme was significantly higher in patients than in controls. It is worth noting here that the percentage of NK and CD56+T cells expressing granzyme B was already very high in control individuals (around 90%). However, in contrast to granzyme A, the MFI of cells expressing granzyme B did not significantly increase, and were even decreased in CD56+T cells. Regarding granzyme K, which is thought to stimulate monocytic cells to secrete pro-inflammatory mediators like granzyme A, our results do not indicate a role for this granzyme in these patients. Further studies are needed to confirm these findings and see whether granzymes are indeed playing a role in the host defense against *Salmonella* Typhi.

In summary, patients demonstrated a marked increase of extracellular levels of granzyme A and B in acute phase plasma of patients with typhoid fever, and showed evidence for an association of these granzymes with higher levels of IFN- γ and with disease severity. Lymphocytes of typhoid fever patients showed higher levels of intracellular granzyme A and B, but not K, compared to healthy controls.
